# The Extent of Eating Disorders and Comorbid Psychopathology Among Adolescent School Pupils

**DOI:** 10.1002/erv.70044

**Published:** 2025-11-03

**Authors:** Sophie Fletcher, Tabitha Jackson, Talar R. Moukhtarian, Carla Toro, Glenn Waller, Caroline Meyer

**Affiliations:** ^1^ Mental Health Innovations Group Warwick Applied Health Warwick Medical School University of Warwick Coventry UK; ^2^ Department of Psychology University of Sheffield Sheffield UK; ^3^ Coventry and Warwickshire Partnership NHS Trust Coventry UK

**Keywords:** adolescents, comorbidity, eating disorders, risk

## Abstract

**Objective:**

Limited literature exists on the extent of eating concerns among adolescents. This study examines the extent of eating disorder pathology and psychosocial correlates among 11‐ to 18‐year‐olds.

**Method:**

School pupils (*N* = 382; 52% female; 72.8% Caucasian) provided demographic information and completed measures of eating disorder pathology (using a cut‐off of > 3.64 on the seven‐item Eating Disorders Examination‐Questionnaire), psychosocial impairment, body shape dissatisfaction and mood. Levels of comorbid problems were compared across adolescents with low‐ and high‐risk for eating disorders, using Mann‐Whitney tests and Chi‐squared tests and an alpha of 0.001 (to account for exploratory analyses).

**Results:**

A fifth (20.7%) of pupils exhibited clinical levels of eating disorder pathology, and they scored significantly worse on the other measures of psychopathology than those without such eating concerns. The majority (89.9%) of pupils with eating disorder pathology scores were above the clinical threshold in one or more comorbid areas. Eating disorder pathology and measures of comorbidity were all significantly intercorrelated.

**Discussion:**

A fifth of pupils were at‐risk of eating disorder pathology, and almost all demonstrated substantial comorbidity. Contrary to the ‘white female’ eating disorder stereotype, many of those with eating concerns were non‐white and over a third did not identify as female. These findings require further work on the screening technology, but highlight the pressing need for access to eating disorder prevention and treatment for a diverse population of such adolescents.

## Highlights

- One fifth of adolescents demonstrated clinical levels of eating disorder pathology
- Most adolescents (∼90%) with eating disorder pathology exhibited substantial psychosocial and mood‐related comorbid concerns
- Diverse risk profile: A significant portion of at‐risk adolescents were non‐white and over one‐third did not identify as female challenging traditional stereotypes

## 1 Introduction

Early identification of risk has been determined to be a key issue when developing and offering interventions for eating disorders (e.g., Mills et al. 2024). Identification and response to such risk has potential implications for effective prevention and treatment of eating disorders, as well as common comorbid difficulties, such as anxiety, depression and psychosocial difficulties (e.g., quality of life). Consequently, it is important that early identification of eating disorder risk should be operationalised, particularly among adolescent groups, where incidence is relatively high.

In keeping with the high levels of eating disorders among adolescents (e.g., M. Solmi et al. 2022), the extent of such risk appears to be substantial in this age group. López‐Gil et al. (2023) suggested that risk for eating disorders is present in 22.36% of children and adolescents, with a higher risk level among the older individuals (i.e., the adolescents). However, this meta‐analysis has the drawback of being based on identified ‘caseness’ using the SCOFF measure, which has poor positive predictive validity (carries the substantial risk of either overestimation or underestimation of cases according to the cut‐off used), making it of questionable use for clinical or screening purposes (F. Solmi et al. 2015; Wan Wahida et al. 2017). Therefore, it is important to consider the potential prevalence of such risk based on research that employs a wider range of measures.

One such meta‐analysis based on a wider set of screening measures is that of Ghazzawi et al. (2023), which concludes that 13.0% of adolescent students can be considered ‘at risk’ of developing eating disorders (defined as scoring above the clinical threshold on screening measures). This prevalence is substantially below that reported using the SCOFF alone (López‐Gil et al. 2023), highlighting the importance of the measure used to identify caseness.

However, the Ghazzawi et al. (2023) meta‐analysis remains limited in extent, with few studies from many of the countries considered, including only one from the United Kingdom. Such studies usually provide minimal information regarding ethnicity, limiting the generalisability of such findings. Finally, eating disorders are commonly comorbid with other conditions (particularly anxiety and depression), meaning that any clinically meaningful understanding of the presentation of eating disorders needs to take such comorbidity into account. Clearly, there is a need for further research into understanding the phenotype and extent of eating disorder pathology within diverse and representative adolescent populations, in order to target prevention and intervention work.

Given the importance of early identification through screening, there is a need to add to research that identifies levels of eating and body image concerns within young people of a potentially vulnerable age (11–18 years). However, to establish the generalisability and clinical utility of such screening, it is important that such research should reflect diversity (ethnicity, age, gender) and comorbid mental health issues. Therefore, the aims of this study were to:

1. Establish the extent of at‐risk eating disorder pathology (i.e., above the clinical cut‐off on an established measure) in a sample of UK adolescents;
2. Determine levels of such risk among sub‐groups (defined by ethnicity, gender, BMI and age);
3. Identify the level of comorbid psychopathology among young people at risk of developing eating disorders.

This was an exploratory pilot study, with the medium‐term aim of determining whether a larger study would be feasible to fix a more robust estimate of likely prevalence in this group, and the longer‐term aim of developing and targeting an intervention to address such cases. While there were no a priori hypotheses for the first or second aim, it was hypothesised that the level of eating disorder risk would be dimensionally and positively associated with levels of comorbid psychopathology (aim 3).

## 2 Method

The study received ethical approval from the Biomedical and Scientific Research Ethics committee, University of Warwick (BSREC 77/22‐23). The study was not pre‐registered.

### 2.1 Participants

Secondary schools (*N* = 66) across the Midlands region of the UK were invited via email to participate in the study, between May and October 2023. To ensure that the pupil populations captured covered a range of socioeconomic backgrounds, the Indices of Multiple Deprivation (IMD) decile score was noted for each school invited, according to their postcode (0 = most deprived areas, 10 = least deprived areas; Ministry of Housing and Communities and Local Government 2019). The IMD decile provides weighted information regarding seven domains of deprivation in relatively small areas in England (e.g., income deprivation, employment deprivation). Use of the IMD decile score is more likely to reflect the experiences of the pupils than other measures of socioeconomic status (e.g., parental income). Fifteen schools expressed interest, with five contributing to the final sample, along with attendees recruited from a local Youth group of attendees at local schools. The recruitment flowchart of school and all schools/youth group characteristics are detailed in Supporting Information S1: Figure S1 and Table S1 respectively. A total of 382 school pupils completed the survey.

### 2.2 Procedure

In each participating school, a local coordinator facilitated the recruitment of participants for the survey. Information about the survey along with participant information leaflet and consent forms (detailed in Supporting Information S1: Figure S2) was shared via school assemblies and online platforms, inviting pupils to take part. For pupils under the age of 16, an opt‐out letter was sent to their parents, containing information about the survey and the one‐week opt‐out window.

After providing informed consent, pupils were asked to complete an anonymous 25‐min questionnaire via the Qualtrics platform, including sociodemographic questions and measures of eating and body image concerns, psychosocial impairment and mood. Survey completion setting was variable depending on school and pupils' age, but predominantly took place during Information Technology lessons or outside of school hours. At the end of the survey, pupils were provided with signposting resources for support in case of any concerns being raised.

It was made clear that survey participation would require completion of all four psychometric measures in full, but that it would not be mandatory for participants to complete some of the sociodemographic information (weight and height), to reduce potential concerns about identifiability or distress. Therefore, while the psychometric data base was complete, there were some missing data on the sociodemographic information.

### 2.3 Measures

Four standardised, validated and age‐appropriate questionnaires were used to measure: eating pathology (Eating Disorder Examination‐Questionnaire 7‐item, EDE‐Q7; Bang et al. 2023; Grilo et al. 2013; Jenkins and Davey 2020); psychosocial impairment (Clinical Impairment Assessment, CIA; Bohn and Fairburn 2008); body dissatisfaction (Body Satisfaction Questionnaire, BSQ; Evans and Dolan 1993); and mood (Depression, Anxiety and Stress Scale‐Youth, DASS‐Y; Szabo and Lovibond 2022). Each measure is well‐validated and has established clinical cut‐offs (see Supporting Information S1: Table S2 for further details on measures and cut‐offs). Sociodemographic questions included age, school year, gender and ethnicity.

Self‐reported weight and height were also optionally collected to calculate weight for height metrics (Body Mass Index—BMI; BMI‐for‐age percentile). The optional nature of providing this information was a result of ethical recommendations that the young people might find this information distressing to provide, and that it would not be possible to support them in that circumstance, as the survey was completed without support. The result was that only a third of participants provided weight and height data, limiting the conclusions that can be reached relating to BMI status in this study.

### 2.4 Data Analysis

Data were analysed using SPSS v.28. Completer analyses were used throughout, given the complete nature of the psychometric database. However, in order to ensure that missing data on socio‐demographic variables did not bias outcomes, multiple imputation was used for secondary intention‐to‐treat analysis (SPSS multiple imputation, with 20 imputations to ensure reliable outcomes) in the case of analysis involving BMI or BMI percentile. Descriptive statistics were used to summarise participant characteristics and clinical presentations. Where the *N* in cells was low enough (usually < 10) to raise the risk of participants being identifiable, cells were concatenated accordingly (e.g., ‘obese’ and ‘overweight’ being treated as a single group). As the dimensional measures were non‐normally distributed, Spearman's correlation coefficients, Mann‐Whitney tests and Chi‐squared tests were used to determine patterns of associations and differences. Effect sizes were calculated for the Mann‐Whitney tests (*tau*) and for the Chi‐squared tests (*phi* for 2 × 2 contingency tables and Cramer's *V* for larger tables), with all effect sizes interpreted using the convention of 0.1 = small, 0.3 = moderate, and 0.5 = large. As the data analysis involved multiple, exploratory comparisons and correlations, a more conservative alpha level was adopted (*p* < 0.001) to reduce the risk of Type 1 errors. For the multiple imputation data, means for BMI and BMI centile were presented, and differences between those with higher versus lower EDE‐Q7 scores were calculated using *t*‐tests with Cohen's *d* to demonstrate effect sizes. Cohen's *d* was interpreted as 0.2 = small effect, 0.5 = medium effect, and 0.8 = large effect.

## 3 Results

In keeping with the completeness of the psychometric data, completer data analyses are presented unless it is stated that the analyses were intention‐to‐treat (ITT).

### 3.1 Levels of Eating Pathology (Aim 1)

Table 1 shows the pupils' sociodemographic characteristics and clinical presentation (mean scores; percentage in each severity category) on the four key measures. Approximately half (52.1%) of participants were female and 72.8% were white, with a mean age of 14.27 years (SD = 1.56; range 11–18). The mean BMI among all pupils who self‐reported height and weight was 21.03 (*n* = 136, SD = 3.62) [ITT mean = 20.18, SD = 3.91]. Regarding BMI‐for‐age percentiles (*n* = 126, male or female), 72.2% were within a healthy BMI range, 15.9% were categorised as overweight, 6.3% as obese and 5.6% as underweight [mean = 54.33; SD = 29.33; ITT mean = 52.85.18, SD = 30.6]. Intention‐to‐treat outcomes were very similar to those from the completer analyses, suggesting no systematic loss of data that could have biased the outcomes.

**TABLE 1:** Participant characteristics (*N* = 382).

Characteristics	Mean (SD)	Frequency *n* (%)
Age^a^	14.27 (1.56)	—
School year		
Year 7 (11–12 years)	—	38 (9.9)
Year 8 (12–13 years)	—	12 (3.1)
Year 9 (13–14 years)	—	214 (56.0)
Year 10 (14–15 years)	—	50 (13.1)
Year 12 (16–17 years)	—	38 (9.9)
Year 13 (17–18 years)	—	30 (7.9)
Gender		
Female	—	199 (52.1)
Male	—	163 (42.7)
Other	—	26 (5.3)
Ethnicity		
Arab, Asian or Asian British	—	32 (8.4)
Black or Black British	—	21 (5.5)
Mixed ethnic groups	—	32 (8.4)
Other	—	19 (5.0)
White	—	278 (72.8)
BMI^b^	21.03 (3.62)	—
BMI‐for‐age percentile^c^	54.33 (29.33)	—
BMI‐for‐age percentile classification^c^		
Underweight/healthy weight	—	98 (77.8)
Overweight/obese	—	28 (22.2)
Eating disorder pathology (EDE‐Q7)		
EDE‐Q7 global score	1.91 (1.82)	—
EDE‐Q7 subscales		
Dietary restraint	1.65 (2.06)	—
Shape/weight overvaluation	2.02 (2.01)	—
Body dissatisfaction	2.05 (2.10)	—
EDE‐Q7 caseness		
Above‐threshold case	—	79 (20.7)
Sub‐threshold case	—	303 (79.3)
Psychosocial impairment (CIA)		
CIA global score	9.62 (12.15)	—
CIA caseness		
Above‐threshold case	—	98 (25.7)
Sub‐threshold case	—	284 (74.3)
Body shape dissatisfaction (BSQ)		
BSQ total score	18.49 (10.98)	—
BSQ caseness		
Above‐threshold case	—	94 (24.6)
Sub‐threshold case	—	288 (75.4)
Mood^d^ (DASS‐Y)		
Depression	4.49 (6.17)	—
Symptom severity		
Normal	—	274 (71.7)
Mild	—	20 (5.2)
Moderate	—	36 (9.4)
Severe	—	23 (6.0)
Extremely severe	—	29 (7.6)
Anxiety	4.32 (5.43)	—
Symptom severity		
Normal	—	271 (70.9)
Mild	—	25 (6.5)
Moderate	—	43 (11.3)
Severe	—	22 (5.8)
Extremely severe	—	21 (5.5)
Stress	7.72 (6.66)	—
Symptom severity		
Normal	—	266 (69.6)
Mild	—	27 (7.1)
Moderate	—	38 (9.9)
Severe	—	22 (5.8)
Extremely severe	—	29 (7.6)
Total negative affect	16.53 (16.98)	—
Symptom severity		
Normal	—	275 (72.0)
Mild	—	19 (5.0)
Moderate	—	40 (10.5)
Severe	—	18 (4.7)
Extremely severe	—	30 (7.9)

Abbreviations: BMI, body mass index; BSQ, body satisfaction questionnaire; CIA, clinical impairment assessment; DASS‐Y, depression, anxiety and stress scale—youth; EDE‐Q7, the eating disorders examination—questionnaire 7‐item.

^a^ *N* = 381, 1 outlier removed.

^b^ *N* = 136, self‐reported height and weight were optional measures.

^c^ *N* = 126, excluding individuals identifying as non‐binary, other or prefer not to say who self‐reported height and weight. This is a noted limitation of the study that gender identity was used to calculate BMI‐for‐age percentiles, instead of sex assigned at birth.

^d^ Szabo and Lovibond (2022) derived severity cut‐offs to categorise the range of scores in a population, whereby “mild” represents a person scoring above the population mean but below the severity of individuals seeking support. For further information see Lovibond and Lovibond 1995. Retrieved from https://dass.psy.unsw.edu.au/DASSFAQ.htm.

Approximately a fifth (20.7%) of participants exhibited at‐risk ED pathology (EDE‐Q7 global score ≥ 3.64). Slightly more (25.7%) experienced clinically significant psychosocial impairment, impacting their overall quality of life (CIA global score ≥ 16), and 24.6% had clinically significant concerns with their body shape (BSQ total score ≥ 26). In addition, between a quarter and a third of participants experienced mild to extremely severe symptoms of depression (28.3%), anxiety (29.1%), stress (30.4%), and total negative affect (28.0%) (DASS‐Y).

### 3.2 Sociodemographic Characteristics of Those With High Risk Levels of Eating Pathology (Aim 2)

Table 2 shows the sociodemographic characteristics of those adolescents with high versus low levels of risk on the EDE‐Q7. The only reliable differences were that those in the high‐risk group were older and had a higher BMI than those in the low‐risk group, though both differences were only based on small effect sizes. Other demographic characteristics did not differ reliably across groups.

**TABLE 2:** Sociodemographic characteristics of low‐risk and high‐risk cases for eating disorder pathology.

	Low‐risk group (*Ν* = 303)	High‐risk group (*n* = 79)	Test statistic	Effect sizes
Sociodemographics				
Age (*M*, SD)^a^	14.12 (1.51)	14.82 (1.64)	*z* = 3.72*	*tau* = 0.10
School year			*X* ^2^ = 23.2*	*V* = 0.12
Year 7–8	41 (12.9)	11 (13.9)		
Year 9	187 (61.7)	27 (34.2)		
Year 10	34 (11.2)	16 (20.3)		
Year 12	21 (6.9)	17 (21.5)		
Years 12 and 13	43 (14.2)	25 (31.6)		
Gender			*X* ^2^ = 11.6	*V* = 0.12
Male	141 (46.5)	22 (27.8)		
Female	150 (49.5)	49 (62.0)		
Other	12 (4.0)	8 (10.1)		
Ethnicity			*X* ^2^ = 3.41	*phi* = 0.09
White	214 (70.6)	64 (81.0)		
Other	89 (29.4)	15 (19.0)		
BMI (*M*, SD)^b^	20.42 (3.47)	22.39 (3.63)	*z* = 3.18*	*tau* = 0.08
BMI‐for age percentile (*M*, SD)^c^	49.33 (29.09)	66.38 (26.59)	*z* = 2.96	*tau* = 0.08
BMI‐for‐age‐percentile classification^c^			*X* ^2^ = 2.38	*phi* = 0.14
Underweight/Healthy weight	73 (82.0)	25 (67.6)		
Overweight/Obese	16 (18.0)	12 (32.4)		

*Note:* Effect sizes (tau, phi and Cramer's V)—0.1 = small; 0.3 = moderate; 0.5 = large.

Abbreviation: BMI, body mass index.

^a^ *N* = 381.

^b^ *N* = 136, self‐reported height and weight were optional measures.

^c^ *N* = 126, excluding individuals identifying as non‐binary, other or prefer not to say.

^*^ *p* < 0.001.

The Intention‐to‐treat analyses for BMI and BMI percentile showed similar patterns to those in Table 2. Mean BMI (low‐risk = 19.67, SD = 3.66; high‐risk = 22.11, SD = 4.26) was different across the two groups (*t* = 5.10; *p* < 0.001; *d* = 0.644). Mean BMI percentile (low‐risk = 49.33, SD = 29.25; high‐risk = 66.35, SD = 31.90) was different across the two groups (*t* = 4.52; *p* < 0.001; *d* = 0.571). However, the ITT analysis for BMI percentile was significant in this case, and the effect sizes were moderate for both BMI indices, rather than having the small effects found in the completer analysis.

### 3.3 Association of Eating Disorder Risk With Psychological Characteristics (Aim 3)

Table 3 shows the correlations between all dimensional variables. Eating disorder attitudes (EDE‐Q7) were significantly and strongly associated with psychosocial impairment, body shape dissatisfaction and mood (Spearman's *rho* = 0.56–0.86, *p* < 0.001 in all cases). Each of the other measures were also intercorrelated.

**TABLE 3:** Spearman's correlations between variables.

Variable	1.	2.	3.	4.	5.	6.	7.	8.	9.	10.
1. EDE‐Q7 global score	—									
2. EDE‐Q7 dietary restraint	0.83*	—								
3. EDE‐Q7 shape/weight overvaluation	0.92*	0.61*	—							
4. EDE‐Q7 body dissatisfaction	0.92*	0.63*	0.89*	—						
5. CIA global score	0.86*	0.65*	0.84*	0.87*	—					
6. BSQ total score	0.86*	0.64*	0.85*	0.87*	0.88*	—				
7. DASS‐Y depression	0.64*	0.47*	0.62*	0.68*	0.75*	0.69*	—			
8. DASS‐Y anxiety	0.59*	0.43*	0.58*	0.63*	0.72*	0.67*	0.80*	—		
9. DASS‐Y stress	0.56*	0.37*	0.57*	0.60*	0.69*	0.66*	0.80*	0.82*	—	
10. DASS‐Y total negative affect	0.63*	0.44*	0.62*	0.67*	0.76*	0.71*	0.89*	0.91*	0.96*	—

Abbreviations: BSQ, body satisfaction questionnaire; CIA, clinical impairment assessment; DASS‐Y, depression, anxiety and stress scale—youth; EDE‐Q7, the eating disorders examination—questionnaire 7‐item.

^*^ *p* < 0.001 (2‐tailed).

Table 4 shows the association of categorical at‐risk eating pathology status (EDE‐Q7 global scores ≥ 3.64) with scores on the other measures of psychopathology. Those with at‐risk EDE‐Q7 scores scored significantly higher than sub‐threshold cases on all other measures (*p* < 0.001 in all cases), with very large effect sizes in all cases. Considering categorical risk scores (over the earlier stated clinical cut‐off on other measures), 89.9% of at‐risk EDE‐Q7 individuals experienced additional clinically significant concerns in at least one other domain (psychosocial impairment, body shape dissatisfaction, mood).

**TABLE 4:** Comparison of pupils with above‐threshold and sub‐threshold eating concerns.

	EDE‐Q7 above‐threshold cases (*n* = 79)	EDE‐Q7 sub‐threshold cases (*n* = 303)	Test statistic	Effect sizes
Eating disorder pathology (EDE‐Q7) (*M*, SD)				
EDE‐Q7 global score	4.75 (0.74)	1.16 (1.17)	*z* = 13.78*	*tau* = 0.71
Dietary restraint	4.37 (1.72)	0.94 (1.46)	*z* = 11.94*	*tau* = 0.61
Shape/weight overvaluation	4.85 (1.15)	1.28 (1.46)	*z* = 12.70*	*tau* = 0.65
Body dissatisfaction	5.03 (1.06)	1.27 (1.54)	*z* = 12.87*	*tau* = 0.66
Psychosocial impairment (CIA)				
CIA global score (*M*, SD)	26.58 (11.62)	5.20 (7.52)	*z* = 11.96*	*tau* = 0.61
CIA caseness (*n*, %)			*X* ^2^ = 152.79*	*phi* = 0.63
Clinical case	63 (79.7)	35 (11.6)		
Non‐clinical case	16 (20.3)	268 (88.4)		
Body shape dissatisfaction (BSQ)				
BSQ total score (*M*, SD)	33.76 (8.86)	14.51 (7.42)	*z* = 12.08*	*tau* = 0.62
BSQ caseness (*n*, %)			*X* ^2^ = 170.80*	*phi* = 0.69
Clinical case	64 (81.0)	30 (9.9)		
Non‐clinical case	15 (19.0)	273 (90.1)		
Mood (DASS‐Y)				
Depression (*M*, SD)	11.38 (6.75)	2.69 (4.54)	*z* = 9.83*	*tau* = 0.50
Symptom severity (*n*, %)			*X* ^2^ = 111.41*	*V* = 0.38
Normal	21 (26.6)	253 (83.5)		
Mild and moderate	23 (29.1)	33 (10.9)		
Severe and extremely severe	35 (44.3)	17 (5.6)		
Anxiety (*M*, SD)	9.47 (6.29)	2.98 (4.27)	*z* = 8.65*	*tau* = 0.44
Symptom severity (*n*, %)			*X* ^2^ = 86.21*	*V* = 0.34
Normal	25 (31.6)	246 (81.2)		
Mild and moderate	26 (32.9)	42 (13.9)		
Severe and extremely severe	28 (35.4)	15 (5.0)		
Stress (*M*, SD)	13.61 (6.47)	6.18 (5.81)	*z* = 8.17*	*tau* = 0.41
Symptom severity (*n*, %)			*X* ^2^ = 83.58*	*V* = 0.33
Normal	26 (32.9)	240 (79.2)		
Mild and Moderate	20 (25.3)	45 (14.9)		
Severe and Extremely Severe	33 (41.8)	18 (5.9)		
Total negative affect (*M*, SD)	34.46 (18.16)	11.86 (13.14)	*z* = 8.97*	*tau* = 0.46
Symptom severity (*n*, %)			*X* ^2^ = 108.95*	*V* = 0.38
Normal	22 (27.8)	253 (83.5)		
Mild and moderate	24 (30.4)	35 (11.6)		
Severe and extremely severe	33 (41.8)	15 (5.0)		

*Note:* DASS‐Y symptoms categories were merged (Mild & Moderate; Severe & Extremely Severe), due to violation of the assumption that the expected frequency in each cell should be five or more in at least 80% of the cells. Effect sizes (tau, phi and Cramer's V)—.1 = small; 0.3 = moderate; 0.5 = large.

^*^ *p* < 0.001.

## 4 Discussion

This exploratory feasibility study of a diverse population of adolescents aimed to determine the extent of at‐risk levels of eating disorder pathology, and the sociodemographic characteristics and comorbid psychopathology of those at risk in this way. Around a fifth of the current sample exhibited high levels of at‐risk ED pathology ‐ substantially higher than the estimated global figure (one in eight pupils) considered to be at risk of developing eating disorders (Ghazzawi et al. 2023), but similar to community samples in Norway (15% at risk of EDs, using the EDE‐Q7; Bang et al. 2023). The majority of those with at‐risk ED pathology in the current study also exhibited marked psychosocial comorbidities. It is particularly noteworthy that the gender identity and BMI of those pupils exhibiting at‐risk ED pathology in the present sample differed from those of the low‐risk group (using completer and intention‐to‐treat analyses, where appropriate), while other characteristics did not differ in that way.

These data are partially comparable with those of the wider population where recruitment took place, which future research should note when considering generalisability of the findings. The obesity and overweight levels identified here (22%) are lower than population statistics for this region of the UK would suggest (obesity alone standing at 24% among the younger group (https://fingertips.phe.org.uk/static‐reports/health‐trends‐in‐england/West_Midlands/obesity.html), without accounting for the overweight group). Considering ethnicity, the most recent UK Government Census (2021) identified that 77%–85.7% of the population of this region of the UK belong to White ethnic groups. The range is relatively similar to the 72% in this study, suggesting that the study reflected local ethnic diversity as well as having a representative gender split and a relatively low socioeconomic status. Therefore, we have shown that the severity of eating concerns and comorbidities is not limited to white females from more affluent households, as stereotypes tend to suggest.

These findings confirm that at‐risk levels of eating disorder pathology are concerningly high and are associated with comorbid mood, body image and quality of life issues, in a relatively representative, diverse population. Such findings suggest that assessment, prevention and treatment of eating and comorbid pathologies should be focused on this younger ‘at risk’ group.

However, it is important to consider the limitations of this study and of the field in general. Recruitment of schools and of participant pupils was sub‐optimal, with only 382 (6.5%) taking part out of the potential 5896 participants (Supporting Information S1: Table S1). This under‐recruitment results in a potential self‐selection bias (Ghazzawi et al. 2023), suggesting that the period of school recruitment should be more carefully planned to avoid examinations, vacations, etc. Crucially, due to the low participation rate, the sample may not be representative of the wider pupil population. As such, findings should not be interpreted as indicative of population prevalence of eating problems in school settings. Instead, the study's strength lies in exploring associations between variables such as sex, age, BMI, and comorbid symptoms within the responding sample. In addition, it proved hard to access parental opt‐out data, meaning that the reasons for non‐participation are unclear. A recent study of non‐participation in school‐based mental health research suggested that implementing stigma reduction strategies, introducing incentives, and close consultation with staff and parents could also improve participation rates (Baldofski et al. 2024) and thus counter potential bias. Future research should also consider collecting IMD decile information for the individual school pupils, rather than by school, to ensure more accurate socioeconomic characterisation of the sample. Alternatively, Boliver et al. (2022) suggest classifying children in this way on the basis of receipt of free school meals, as that index results in fewer false positives for social deprivation. Such research could also collect additional deprivation data based on where the child lives (as implemented by the NHS National Child Measurement Program). Other limitations are the lack of complete BMI data for the full sample and the reliance on self‐reporting, which are likely to require supported in‐person weighing to overcome potential distress (see Method).

### 4.1 Implications and Future Directions

As outlined in the Introduction, this was an exploratory pilot study designed to assess the feasibility of conducting research on eating pathology in school‐based settings. In line with this, our findings provide early signals regarding patterns of associations between eating concerns and demographic and psychological variables, rather than robust estimates of prevalence. The limitations discussed above, particularly the low response rate and potential selection bias, reinforce the need for future studies with improved recruitment strategies and larger, more representative samples to accurately estimate prevalence and inform intervention development.

In terms of public health, these findings clearly indicate that obesity and overweight are as much of a problem in the United Kingdom as they are in other countries (e.g., Ghazzawi et al. 2023), and therefore need planning around healthy eating and prevention. However, they also show that the health implications of obesity extend to linked phenomena of unhealth eating attitudes, body image, mood, and quality of life. Therefore, health policy and practice should include clinical provision for each of these psychosocial disturbances.

Future research in this field will be dependent on the development of better‐validated screening measures of eating pathology among adolescents, including age‐relevant cut‐off scores (Feltner et al. 2022; Ghazzawi et al. 2023; House et al. 2022) and ethnically‐appropriate measures. It will also be important to implement measures that relate to Avoidant/Restrictive Food Intake Disorder (ARFID), as measures such as the EDE‐Q7 are poor at identifying ARFID presentations. Validation through the collection of longitudinal data, as well as diagnoses and treatment status will be an important second stage once such screening is fully validated, allowing prevention work and interventions that could interrupt the severity of eating pathology and its links to depression and anxiety disorders, as well as self‐harm (Micali et al. 2017). Future intervention planning efforts should prioritise the needs of diverse young people and their parents (gender; ethnicity; socioeconomic status) in ways that minimise disruption to educational activities and academic commitments.

## Funding

This work was supported by the Rosetrees Trust [Grant number: Seedcorn2022\100167]. Rosetrees Trust had no role in the study design, collection, analysis or interpretation of the data, writing the manuscript, or the decision to submit the paper for publication.

## Conflicts of Interest

The authors declare no conflicts of interest.

## Supporting information


Supporting Information S1

## Data Availability

For requests to access the anonymous survey dataset, please contact the corresponding author.
