# Supplementary material for: The Extent of Eating Disorders and Comorbid Psychopathology Among Adolescent School Pupils
Source: Eur Eat Disord Rev. 2025 Nov 3;34(3):513–21. doi: 10.1002/erv.70044 (PMC13048745; doi:10.1002/erv.70044)
Supplement: Supplementary file 1 — Supporting Information S1 [file ERV-34-513-s001.docx]

**Supplementary materials**


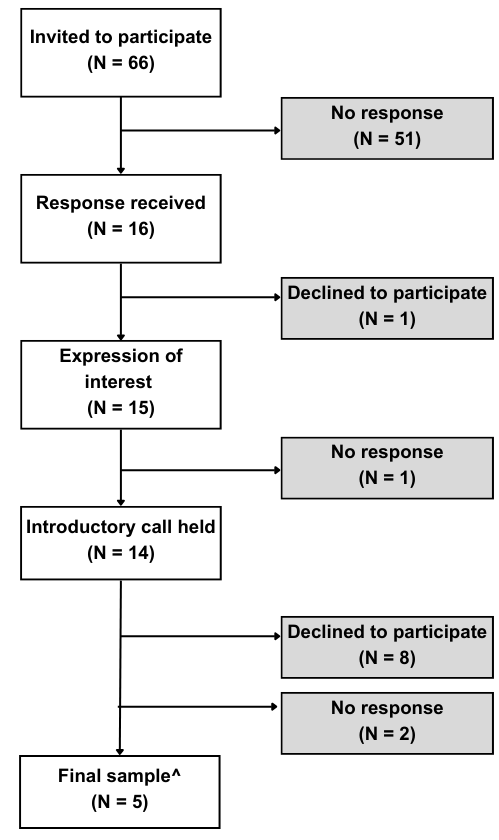
**Supplementary Figure 1.** *Recruitment flowchart*

*Note.* ^ Youth Group included in final sample.

## **Supplementary Table 1.** *School and youth group characteristics*

| **School** | **Type** | **Population (2022/23)^1^** | **IMD Decile Score^2^** |
| --- | --- | --- | --- |
| 1 | State | 1322 | 1 |
| 2 | State | 1017 | 4 |
| 3 | State | 908 | 5 |
| 4 | Grammar | 960 | 7 |
| 5 | State | 1672 | 9 |
| Youth Group | N/A | 17 | 4 |

***References***

1. Department for Education (2023). *Compare the performance of schools and colleges in England*. Retrieved from <https://www.gov.uk/school-performance-tables?_ga=2.139230387.121873245.1715253113-811750178.1712650803>

2. Ministry of Housing, Communities and Local Government. (2019). *English indices of deprivation 2019.* Retrieved from <https://www.gov.uk/government/statistics/english-indices-of-deprivation-2019>

**Supplementary Figure 2.** *Participant Information Leaflet and consent form for young people*

**
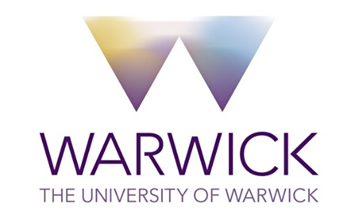
**

| **Study Title:** | Ensuring rapid access to brief, effective treatment for adolescents with eating and body concerns: Determining the feasibility of an intervention programme in schools |
| --- | --- |
| **Investigator(s):** | Talar Moukhtarian (Warwick Medical School), Caroline Meyer (Warwick Medical School), Carla Toro (Warwick Medical School), Sophie Tyerman (Warwick Medical School), Charlotte Kershaw (Warwick Medical School), Glenn Waller (University of Sheffield). |

**Introduction**

We are inviting you to take part in a research study. Before you decide to say yes or no, you need to understand why we are doing the research, and what it would involve for you. Please take the time to read the leaflet carefully. We encourage you to talk to your parent/carers about the study, and to others if you wish.

Please ask us if there is anything that is not clear, and take time to decide whether or not you want to take part.

**Who is organising and funding the study?**

This project is funded by the Rosetrees Trust. The design, implementation and management of this study is being conducted by researchers at the Warwick Medical School at the University of Warwick.

**What is the study about?**

We know that a therapy called CBT-T, which involves recommendations for healthy eating and learning to respond to feelings of hunger and fullness, works for people who have troubles with their eating and feel bad about how their body looks (body image). It can be hard to fit in this therapy with going to school, which may mean people don’t take part. We would like to understand if this therapy could be provided to children and young adults at school, making it more available to people who need it without having to travel to a specialist service. We are doing this work at several different schools in the West Midlands area of the UK.

To find out if this is something that could be done, and learn about how it could be done, we will talk to different people who would be involved in either offering the therapy or taking part. As one part of this, we are sending out online surveys to young people (11-18 year olds) to understand how many people are worried about their eating or how their body looks, and how likely it is for them to take part in therapy offered in schools.

**What would taking part involve?**

Taking part in this study requires you to complete an online survey questionnaire, which you will be redirected to at the end of this form.

The survey has four sections-

1. Consent form that you need to complete to access the questionnaire.
2. Questions about yourself (e.g. age, ethnicity, gender).
3. Questions about eating and body image concerns, quality of life and mood.

It will take you on average 10 minutes to complete the questions.

**Do I have to take part?**

No. Participation is completely up to you, and choosing not to take part will not affect you or your education at your school in any way. You can also change your mind after choosing to take part, without giving a reason. Further details about withdrawing from the study are provided later on in this document.

**What are the possible benefits of taking part in this study?**

There are no direct or immediate benefits to you taking part, but by sharing your views you could help make this therapy available to young people who need it in the future.

**What are the possible disadvantages, side effects or risks, of taking part in this study?**

We do not anticipate any risks or disadvantages from taking part in this study. But completing the online survey means reflecting on your own thoughts and feelings about eating, body image and mood, and this may make you feel anxious. We will make sure to offer support. This will include contact details for the eating disorder charity Beat (https://www.beateatingdisorders.org.uk/get-information-and-support/get-help-for-myself/i-need-support-now/) and the contact details of the research team (please see email addresses for the lead researcher and research assistant below).

**Expenses and payments**

There will not be any incentives or rewards to take part in this study.

**Who will know that I’ve taken part?**

If you are under 16 then we will have asked your parent or caregiver to consent to your taking part in the study. If you then decide not to take part, that will be completely up to you.

We will not ask you to tell us your name or any other information that can identify you. We will ask you to answer some questions about your age, ethnicity, mood and eating and body image concerns, and use this anonymous data in reports and publications. However it will not be possible to identify you.

**What will happen to the data collected about me?**

We will be using information from you in order to undertake this study and will act as the data controller for this study. The University of Warwick will keep information collected from this study for 10 years after the study has finished.

No identifiable data such as your name will be collected from you as part of this study. This means that once you complete the online survey, it will not be possible to go back and delete this data as your individual responses cannot be traced back to you.

**Data Sharing**

Your rights to access, change or move your information are limited, as we need to manage your information in specific ways in order for the research to be reliable and accurate. The University of Warwick has in place policies and procedures to keep your data safe.

This data may also be used for future research, including impact activities following review and approval by an independent Research Ethics Committee and subject to your consent at the outset of this research project.

For further information, please refer to the University of Warwick Research Privacy Notice which is available here: <https://warwick.ac.uk/services/idc/dataprotection/privacynotices/researchprivacynotice> or by contacting the Legal and Compliance Team at [infocompliance@warwick.ac.uk](mailto:infocompliance@warwick.ac.uk).

**What will happen if I don’t want to carry on being part of the study?**

You can change your mind about taking part at any point, and this would not affect you or your education in any way. You can close the survey (close the webpage) at any point and this will end your participation and your data will not be saved.

Please note that if you withdraw from the study after you complete the online survey, it will not be possible to delete your data which have already been collected because they have been anonymised. To safeguard your rights, we will keep the data secure in line with the University’s Information and Data Compliance policies*.*

**What will happen to the results of the study?**

After this study we hope to have enough data to be able to ask Rosetrees Trust for more funding to run a trial providing the therapy in schools. We will also report back to Rosetrees what we find out about how the therapy could be provided in schools, and how young people, parents, and school staff feel about it.

**Who has reviewed the study?**

This study has been reviewed and given favourable opinion by the University of Warwick’s Biomedical & Scientific Research Ethics Committee (BSREC): BSREC 77/22-23 (11/04/2023).

**Who should I contact if I want further information?**

If you want further information or have any questions or concerns, please contact lead researcher Dr. Carla Toro at carla.toro@warwick.ac.uk, or research assistant Sophie Tyerman at sophie.tyerman@warwick.ac.uk.

**Who should I contact if I wish to make a complaint?**

Any complaints you have will be taken seriously. Please send any complaints to the person below, who is a senior University of Warwick official entirely independent of this study:

**Head of Research Governance**

Research & Impact Services

University House

University of Warwick

Coventry

CV4 8UW

Email: [researchgovernance@warwick.ac.uk](mailto:researchgovernance@warwick.ac.uk)

Tel: 02476 575733

If you wish to raise a complaint on how we have handled your personal data, you can contact our Data Protection Officer who will investigate the matter: DPO@warwick.ac.uk.

If you are not satisfied with our response or believe we are processing your personal data in a way that is not lawful you can complain to the Information Commissioner’s Office (ICO).

**Thank you for taking the time to read this Participant Information Leaflet**

**Please select the boxes if you agree with the below statements. You will then be redirected to complete the survey.**

| 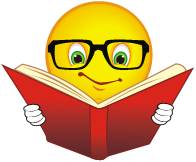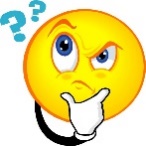 | I have listened to and understand the information about the study, I know I can ask questions and to not take part at any point |  |
| --- | --- | --- |
| 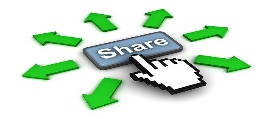 | I am happy for the information I give to be shared with the project team at University of Warwick and used again in the future |  |
| 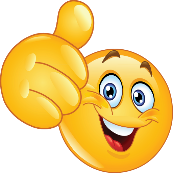 | I agree to take part in the online survey |  |

## **Supplementary Table 2.** *Descriptions, scoring and interpretation information, clinical cut-off or severity cut-off scores and psychometric information for the four outcome measures*

| **Outcome** | **Measure and reference** | **Description** | **Scoring and interpretation** | **Clinical/severity cut-off score** | **Psychometrics** |
| --- | --- | --- | --- | --- | --- |
| Eating disorder pathology  (Primary outcome variable) | The Eating Disorders Examination – Questionnaire 7-item^1^ | A 7-item measure of disordered eating behaviours and attitudes over the past 28 days | Three mean subscale scores (dietary restraint, shape/weight overvaluation, body dissatisfaction) and mean global score; subscale and global score ranges 0-6; higher scores indicate greater severity or frequency of disordered eating behaviours and attitudes | Global score ≥ 3.64^2^ | Good internal consistency (Raykov’s rho of each subscale > .86^1^, McDonald's ω of each subscale > .85^3^; Cronbach α > .89^4^) |
| Psychosocial impairment | Clinical Impairment Assessment (CIA)^5,6^ | A 16-item measure of the severity of psychosocial impairment secondary to eating disorder features over the past 28 days | Global sum score; score range 0-48; higher scores indicate higher level of psychosocial impairment | Global score ≥ 16 ^5,7,8,9^ | Good internal consistency (Cronbach α = .97^5^; Cronbach α = .95^7;^ Cronbach α = .98^8^; Cronbach α = .94^9^) |
| Body shape dissatisfaction | Body Satisfaction Questionnaire (BSQ)^10^ | An 8-item measure of concerns with body shape over the past 28 days | Total sum score (range 0-48); higher scores indicate greater body dissatisfaction | Total score ≥ 26 ^11,12^ | Good internal consistency (Cronbach α > .93^11^; Cronbach α > .91^10^; Cronbach α > .94^12^) |
| Mood | Depression, Anxiety and Stress Scale-Youth (DASS-Y)^13^ | A 21-item measure of negative emotional states over the past 7 days | Three sum subscale scores (depression, anxiety, stress) and total score of negative affect; subscale scores range 0-21; total score range 0-63; higher scores indicate more severe symptoms | Depression: normal = 0-6; mild = 7-8; moderate = 9-13; severe = 14-16;  Extremely severe = 17-21^13^ | Good internal consistency (Cronbach α of each subscale > .84) ^13^ |
|  |  |  |  | Anxiety: normal = 0-5; mild = 6-7; moderate = 8-12; severe = 13-15; extremely severe = 16-21^13^ |  |
|  |  |  |  | Stress: normal = 0-11; mild = 12-13; moderate = 14-16; severe = 17-18; extremely severe = 19-21^13^ |  |
|  |  |  |  | Total negative affect: normal = 0-23; mild = 24-29; moderate = 30-39; severe = 40-46; extremely severe = 47-63 ^13^ |  |

**References**

1. Grilo, C. M., Henderson, K. E., Bell, R. L., & Crosby, R. D. (2013). Eating disorder examination-questionnaire factor structure and construct validity in bariatric surgery candidates. *Obesity Surgery*, **23**(5), 657–662. <https://doi.org/10.1007/s11695-012-0840-8>
2. Bang, L., Nordmo, M., Nordmo, M., Vrabel, K., Danielsen, M., & Rø, Ø. (2023). Comparison between the brief seven-item and full eating disorder examination-questionnaire (EDE-Q) in clinical and non-clinical female Norwegian samples. *Journal of Eating Disorders, 11*(1), 194. <https://doi.org/10.1186/s40337-023-00920-x>
3. Jenkins, P. E., & Davey, E. (2020). The brief (seven‐item) eating disorder examination‐questionnaire: Evaluation of a non‐nested version in men and women. *International Journal of Eating Disorders*, *53*(11), 1809-1817. <https://doi.org/10.1002/eat.23360>
4. Grilo, C. M., Reas, D. L., Hopwood, C. J., & Crosby, R. D. (2014). Factor structure and construct validity of the eating disorder examination‐questionnaire in college students: Further support for a modified brief version. *International Journal of Eating Disorders*, *48*(3), 284-289. <https://doi.org/10.1002/eat.22358>
5. Bohn, K., Doll, H. A., Cooper, Z., O’Connor, M., Palmer, R. L., & Fairburn, C. G. (2008). The measurement of impairment due to eating disorder psychopathology. *Behaviour Research and Therapy*, *46*(10), 1105–1110. <https://doi.org/10.1016/j.brat.2008.06.012>
6. Bohn, K., & Fairburn, C. G. (2008). Clinical impairment assessment questionnaire (CIA 3.0). In C. G. Fairburn (Ed.), *Cognitive behavior therapy and eating disorders* (pp. 315–318). New York, NY: Guilford Press.
7. Calugi, S., Dalle Grave, A., Chimini, M., Lorusso, A., & Dalle Grave, R. (2024). Illness duration and treatment outcome of intensive cognitive‐behavioral therapy in adolescents with anorexia nervosa. International Journal of Eating Disorders.
8. Knatz Peck, S., Towne, T., Wierenga, C. E., Hill, L., Eisler, I., Brown, T., Han, E., Miller, M., Perry, T., & Kaye, W. (2021). Temperament-based treatment for young adults with eating disorders: acceptability and initial efficacy of an intensive, multi-family, parent-involved treatment. *Journal of Eating Disorders, 9*, 1-17. <https://doi.org/10.1186/s40337-021-00465-x>
9. Reas, D. L., Rø, Ø., Kapstad, H., & Lask, B. (2009). Psychometric properties of the clinical impairment assessment: norms for young adult women. *International Journal of Eating Disorders, 43*(1), 72-76. <https://doi.org/10.1002/eat.20653>
10. Evans, C., & Dolan, B. (1993). Body shape questionnaire: Derivation of shortened “alternate forms”. *International Journal of Eating Disorders*, *13*(3), 315–321. [https://doi.org/10.1002/1098-108X(199304)13:3<315::AID-EAT2260130310>3.0.CO;2-3](https://doi.org/10.1002/1098-108X(199304)13:3%3c315::AID-EAT2260130310%3e3.0.CO;2-3)
11. Edlund, K., Johansson, F., Lindroth, R., Bergman, L., Sundberg, T., & Skillgate, E. (2022). Body image and compulsive exercise: are there associations with depression among university students? *Eating and Weight Disorders-Studies on Anorexia, Bulimia and Obesity, 27*(7), 2397-2405. <https://doi.org/10.1007/s40519-022-01374-x>
12. Mueller, V. M., Forrer, F., & Munsch, S. (2024). Psychological correlates of body dissatisfaction in Swiss youth over a one-year study-period. *Frontiers in Psychology, 14*, 1269364. <https://doi.org/10.3389/fpsyg.2023.1269364>
13. Szabo, M., & Lovibond, P. F. (2022). Development and psychometric properties of the DASS-Youth (DASS-Y): An extension of the Depression Anxiety Stress Scales (DASS) to adolescents and children. *Frontiers in Psychology*, *13*, 766890. <https://doi.org/10.3389/fpsyg.2022.766890>
